# Good validity in the Norwegian Knee Ligament Register: assessment of data quality for key variables in primary and revision cruciate ligament reconstructions from 2004 to 2013

**DOI:** 10.1186/s12891-022-05183-2

**Published:** 2022-03-09

**Authors:** Espen Midttun, Morten Torheim Andersen, Lars Engebretsen, Håvard Visnes, Anne Marie Fenstad, Jan-Erik Gjertsen, Andreas Persson

**Affiliations:** 1 Department of Neurology, Førde Central Hospital, Svanehaugvegen 2, 6812 Førde, Norway; 2 Medical Department, Haraldsplass Deaconess Hospital, Bergen, Norway; 3 Department of Orthopaedic Surgery, Oslo University Hospital, Oslo, Norway; 4 Oslo Sports Trauma Research Center, Norwegian School of Sport Sciences, Oslo, Norway; 5 Department of Orthopaedic Surgery, The Norwegian Knee Ligament Register, Haukeland University Hospital, Bergen, Norway; 6 Department of Orthopaedics, Sørlandet Hospital Kristiansand, Kristiansand, Norway; 7 The Norwegian National Advisory Unit On Arthroplasty and Hip Fractures, Department of Orthopaedic Surgery, Haukeland University Hospital, Bergen, Norway; 8 Department of Orthopaedic Surgery, Haukeland University Hospital, Bergen, Norway; 9 Department of Clinical Medicine, University of Bergen, Bergen, Norway

**Keywords:** Anterior cruciate ligament, Registries, Data accuracy, Epidemiology, Predictive value

## Abstract

**Background:**

The Norwegian Knee Ligament Register was founded in 2004 to provide representative and reliable data on cruciate ligament surgery. The aim of this study was to evaluate the validity of key variables in the Norwegian Knee Ligament Register to reveal and prevent systematic errors or incompleteness, which can lead to biased reports and study conclusions.

**Method:**

We included a stratified cluster sample of 83 patients that had undergone both primary and revision anterior cruciate ligament surgery. A total of 166 medical records were reviewed and compared with their corresponding data in the database of the Norwegian Knee Ligament Register. We assessed the validity of a selection of key variables using medical records as a reference standard to compute the positive predictive values of the register data for the variables.

**Results:**

The positive predictive values for the variables of primary and revision surgery ranged from 92 to 100% and from 39 to 100% with a mean positive predictive value of 99% and 88% respectively. Data on intraoperative findings and surgical details had high positive predictive values, ranging from 91 to 100% for both primary and revision surgery. The positive predictive value for the variable “date of injury” was 92% for primary surgeries but only 39% for revision surgeries. The positive predictive value for “activity at the time of injury” was 99% for primary surgeries and 52% for revisions.

**Conclusion:**

Overall, the data quality of the key variables examined in the Norwegian Knee Ligament Register was high, making the register a valid source for research.

**Supplementary Information:**

The online version contains supplementary material available at 10.1186/s12891-022-05183-2.

## Background

The most common serious knee injury in the young population is a tear to the anterior cruciate ligament (ACL). Young females are particularly at risk, with the highest incidence found for young female soccer players [1]. In a population-based cohort study in the United States, the yearly incidence of clinical ACL injuries was found to be 68.6 per 100 000 person-years [2], and it is estimated that 50–70% of patients with ACL injuries undergo reconstructive surgery [3]. The general purpose of reconstructive surgery is to restore joint stability and enable patients to return to their pre-injury activity. The quality of the reported literature on patients’ return-to-sport rate is debated [4], but the rate was found to be 82% in a recent meta-analysis and systematic review [5].

Over the past few years, the number of medical registries has increased, giving clinicians and researchers access to large clinical databases. Registries can be used to improve results through feedback to hospitals and surgeons, and can identify prognostic factors and inferior procedures and devices [6]. The Norwegian Knee Ligament Register (NKLR) was founded in 2004 as the world’s first cruciate ligament register [3]. It collects information on primary cruciate ligament reconstructions and subsequent surgery to the index knee in public and private hospitals in Norway. The purpose of the NKLR is to provide representative and reliable data for epidemiological and observational studies and thus improve treatment options and complement available literature to provide high-quality evidence-based care for patients with cruciate ligament injuries.

When defining data quality in medical registries, the two most cited quality attributes are completeness and accuracy [7]. Several quality control procedures have been proposed in order to preserve these attributes [7–9]. Of great importance are prevention of insufficient data quality through clear data definitions, standard guidelines for data collection, and adequate training and motivation of personnel. In addition, detection of inadequate data quality through systematic monitoring and validation of the data can identify and correct systematic reporting and recording errors. This is often done by comparing the register database with data from another independent data source, e.g. national patient registers [10] or medical records [11]. Previous studies to assess the completeness of the NKLR have found registration rates of between 84 and 97% [3, 10, 12]. The accuracy of data in the NKLR has, however, not yet been investigated. Therefore, the objective of the present study was to validate the data quality of key variables in the NKLR, by comparing register data with the patients’ medical records as reference.

## Methods

### The Norwegian Knee Ligament Register

The Norwegian Knee Ligament Register is run by the Norwegian National Advisory Unit on Arthroplasty and Hip Fractures. All primary reconstructions of cruciate ligaments and subsequent surgeries are to be reported to the NKLR. Since 2017 it has been mandatory to report data to the NKLR, but reporting requires informed patient consent. If the patient has signed an informed consent form for research, the surgeon postoperatively records information on a standard one-page paper form that is sent by post to the NKLR for registration [3]. Digital registration via a secure online form has expanded since 2016, and will be implemented at all Norwegian hospitals performing cruciate ligament reconstructions [13].

The data collected for this study have previously been described [3], and include patient-specific data in addition to intraoperative findings and surgical techniques. In addition, during the study period, patients were asked to report on a paper form subjective knee function based on the Knee injury and Osteoarthritis Outcome Score (KOOS) preoperatively and at 2, 5, and 10 years follow-up [3, 14].

### Study population and design

The NKLR was used to identify patients that had undergone both primary and revision anterior cruciate ligament reconstructions between 2004 and 2013, and 935 patients were found to be eligible for inclusion. We assumed that the error frequency was similar in all reporting hospitals. We therefore included a cluster of patients that had had both primary and revision surgery at the same or a nearby hospital in regions easily accessible for data collection. Of the eligible patients, 250 were considered for inclusion. They had surgery in eight different hospitals, both public and private, in two of Norway’s four health regions. An informed consent form for participation was sent by post, and the completed form could be returned either in an enclosed envelope or by e-mail. A reminder was sent to patients that did not respond within six months. All patients received a personal study ID labeling the consent in order to ensure confidentiality. All patients who returned an informed consent form were included in the study.

### Data collection

Each participant was given a study ID, and the key to the patient’s personal ID number was kept on a secure research server and available on a single paper form for the study personnel when they retrieved the data from the medical records. Medical record data retrieval for hospitals in the Western Health Region (Stavanger University Hospital, Haraldsplass Diaconal Hospital, Haukeland University Hospital and Haugesund Hospital) was accessed electronically, whereas two hospitals (Oslo University Hospital and Martina Hansen’s Hospital) had to be visited to retrieve data. In order to ensure that the review protocol was followed, two of the authors that were not involved in the surgical treatment/rehabilitation of the patients (EM and MTA) systematically reviewed the medical records and the data were coupled with the data retrieved from the register in a standard form. Any uncertainty about data definitions in the medical record was discussed with the senior author (AP). Data on the following variables were retrieved for analysis: hospital of primary and revision surgery, date of injury, activity at the time of injury, index knee, date of primary and revision surgery, femoral and tibial fixation implants, graft choice, meniscal lesions (medial, lateral and both menisci), cartilage lesions (International Cartilage Repair Society (ICRS) grade 1–4), reported ligament injuries at the time of surgery (anterior cruciate ligament (ACL), posterior cruciate ligament (PCL), medial collateral ligament (MCL), lateral collateral ligament (LCL) and posterolateral corner (PLC) injuries), and cause of revision for revision surgeries. The investigated variables were chosen either because of their importance as outcome predictors reported in previous studies (graft choice, graft fixation, reported cartilage lesion) [15–17] or at random to ensure a representative sample of the dataset (activity at the time of injury, date variables, cause of revision, index knee, other ligament or meniscal lesions).

### Data definition

Variables not registered in the NKLR database or found in the medical records were defined as missing data in those sources. In cases where variables could be left unregistered and were not entered in the register form, we defined it as the surgeon intentionally omitting them. For the variable “cause of revision”, it was possible for the surgeon to record one or more causes. Cases where the medical records stated two causes of revision, and the NKLR had only registered one of these, or vice versa, were labelled “partly identical” and were not included in the calculation for that variable. For the variable “method of fixation”, cases were labelled “partly identical” and were not included in the calculation for the variable when the patients had undergone both ACL and PCL surgery and the fixation method was registered correctly for only one of the two. The variable “time of injury” was reported to the register by the surgeon as the month of injury, but in the medical records often as the date of injury. Thus, we classified the data as correct if the date of injury found in the medical record was in the month reported by the surgeon to the NKLR.

### Statistics

To validate the data quality of the variables in the NKLR, we used medical records as the reference standard. We computed positive predictive values (PPVs) with 95% confidence interval (CI) according to the Agresti-Coull method [18]. The PPV for each variable was defined as the number of patients with identical values in the NKLR and in the medical record, divided by the total number of patients with the given variable in the NKLR. PPV was computed for the variables for both primary and revision reconstructions.

## Results

**Fig. 1:**
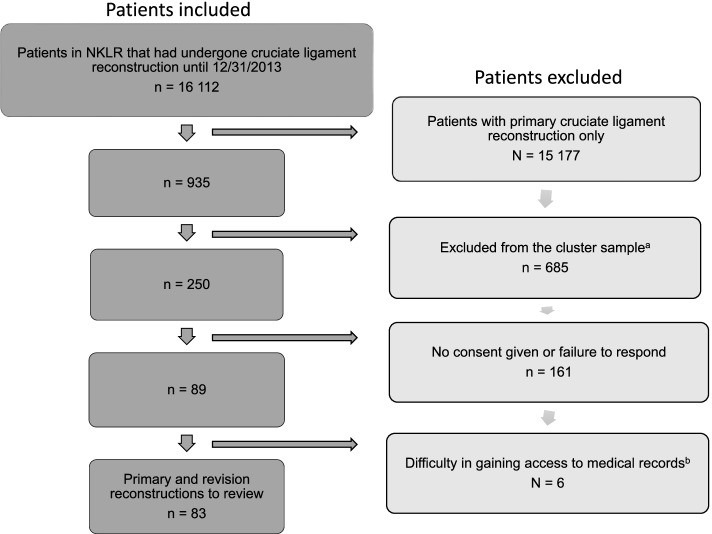
Flowchart of included patients. ^a^ Based on health regions, number of possible patients from each hospital, accessibility to medical records, limited time and resources. ^b^ Due to lack of response from two hospitals. Abbreviation: NKLR, Norwegian Knee Ligament Register

**Fig. 2:**
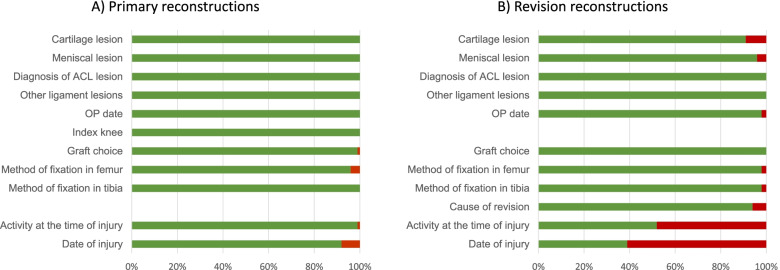
Positive predictive values for investigated variables in primary and revision reconstructions. **A** Positive predictive values for variables in primary reconstructions. **B** Positive predictive values for variables in revision reconstructions. Abbreviations: ACL, anterior cruciate ligament; OP, operation

Eighty-nine patients agreed to participate in the study. One private hospital was excluded due to lack of response to our repeated enquiries for access to the patients’ medical records, thus five patients were excluded. One public hospital was also excluded for the same reason, excluding one patient. Consequently, data from 83 patients were included in the final analysis, giving a total of 166 NKLR forms with corresponding data in the medical records to be reviewed and validated (Fig. 1). The PPVs for the variables of primary and revision surgery ranged from 92 to 100% and 39 to 100% with a mean PPV of 99% and 88% respectively (Additional files 1 and 2). Variables often used in research from the NKLR (meniscal lesions, other ligament injuries, graft choice with graft fixation) had a high PPV (96–100%, Additional file 1). The reported date of injury had the lowest PPV for details reported at primary surgery with a PPV of 92%. Some variables showed a difference in the PPVs for primary and revision surgery (Fig. 2). The PPVs for the variables “date of injury” and “activity at the time of injury” were 92% and 99% for primary surgeries but only 39% and 52% for revision surgeries respectively. For those two variables, the surgeon had reported the same values at revision surgery as were reported in the corresponding primary surgery, leading to 74% (34/46) and 92% (36/39) of the errors respectively. We found that in 25 out of 72 cases where data on “cartilage lesions” were recorded in the patient’s medical record, the corresponding data were not registered in the NKLR. The non-registered cartilage lesions consisted mainly of ICRS grade 1 or 2 lesions (23 out of 25), and occurred more often in the setting of revision surgery (15 out of 25) than in primary surgeries.

## Discussion

The most important finding in this study is that the accuracy of key variables in the NKLR was high, especially for primary reconstructions. Until 2020, data from the NKLR have been used in 71 publications in international journals [13]. Feedback to the surgeons and hospitals is presented in annual reports and at the annual conference of the Norwegian Arthroscopy Association. Findings from studies based on data from the NKLR have caused changes in clinical practice [13, 16, 19], and this study confirms that the underlying data leading to these changes are accurate. However, we did find some recurring errors of registration, where the most frequent errors had occurred in connection with revision surgery.

For most of the investigated variables there were no differences in registration accuracy between primary and revision surgery. The majority of the errors for the variables “activity at the time of injury” and “date of injury” were linked to forms completed after revision surgery. The surgeon often recorded identical information on “activity at the time of injury” and “date of injury” to what had been previously recorded for the primary reconstruction. The reason for this could be that the patient did not have any new major trauma leading up to the revision surgery, and that the surgeon therefore used information from the initial injury. A clear data definition guideline and automatic control of data entry in the digital data forms could potentially have decreased these errors. Due to the low accuracy for the variables “date of injury” (PPV 39%) and “activity at the time of injury” (PPV 52%) for revision surgeries, we recommend that the variables are not included in future research studies. We also recommend that “date of injury” (PPV 92%) in primary reconstructions is interpreted with caution, especially in analysis where this variable is thought to be of clinical importance.

Registration of cartilage lesions showed a high PPV of 100% and 91% for primary and revision surgery respectively. However, superficial cartilage lesions (ICRS grade 1) were found to be underreported to the NKLR compared with the patients’ medical records. In line with our findings, a similar study from the Danish Knee Ligament Reconstruction Register also found missing data on cartilage lesions due to imprecise registration [20]. Possible reasons for the missing data could be that superficial cartilage lesions are common and might not be considered an important finding, or that the lesions would be regarded as a “normal finding” given the age of the patient. We recommend emphasizing to surgeons the importance of registration of all cartilage lesions, as the incidence of superficial cartilage lesions might be higher than previously reported [21]. Several studies from the NKLR have included cartilage lesions in their analysis [22–24], although most of the studies only include deep cartilage lesions (ICRS grade 3 and 4), which in our study proved to have good validity.

Although the “diagnosis of ACL lesion” variable showed a PPV of 100% for both primary and revision surgery, we found that ACL injury was not always recorded on the form following revision surgery. This may have been because there is no guideline for this variable in the form, or simply that the injury was to the ACL graft, and not to the native ACL. Adding a simple guideline to this variable in the form could help prevent these errors in the future.

The “cause of revision” variable showed a satisfactory PPV of 94%. Nevertheless, we discovered that in 16% of the cases the value of the variable was missing in the NKLR. Failure of ACL reconstruction is often multifactorial [25, 26] and the direct cause can often be difficult to determine [27]. In the literature, the categorization of causes of revision is heterogeneous [25, 27–31]. With that in mind, a likely explanation for some of the “missing cases” might be that the surgeon was uncertain of the cause of revision and as a result refrained from filling in this variable altogether. The surgeon can manually report “other cause” of revision in addition to the already printed causes on the paper form. In 4% of the revisions, tunnel misplacement was manually reported as either the only cause of revision or in combination with another cause. Technical errors, especially tunnel misplacement, have previously been reported to be a significant cause of revision [32–35], and tunnel misplacement should therefore be added as an option to the list of causes.

Overall, this study demonstrates high validity of the data in the NKLR. We believe that important reasons for the high data quality are the thorough routines and highly qualified personnel of the Norwegian National Advisory Unit on Arthroplasty and Hip Fractures, with their considerable experience of managing national medical registries. The findings in our study suggest that the NKLR’s instructions to surgeons and data collection protocol are adequate. Routines are in place at the different hospitals to enable surgeons to fill in the form immediately after surgery without any inconvenience, in order to reduce the risk of recall bias. The time required to fill out the form seems to be acceptable, thus giving the register high compliance. The great majority of surgeons have filled out the forms [3, 10, 12], even though this was voluntary during the study period [13]. This suggests that the surgeons are genuinely interested in and motivated to contribute to the research and development of this field of orthopaedic surgery. The annual reports from the NKLR and the yearly update from the NKLR at the annual conferences of the Norwegian Orthopaedic Association and the Norwegian Arthroscopic Association are probably important in contributing to this motivation.

The NKLR is currently in the process of introducing a digital registration form that makes it possible to guide and give immediate feedback to surgeons filling in the form. This will further improve data quality as it allows for easily accessible descriptions of variable definitions, especially some of those found to have a low PPV in this study. Further, digital registration may eliminate missing data since data entry of key variables can be set as mandatory and it is possible to embed immediate automatic control of variables that have a logical association, for instance that the time of surgery comes after the time of injury.

The main strength of this study is its design, where five of the eight clinics included in the cluster were among the large-volume hospitals performing cruciate ligament reconstructions in Norway. Hospitals from the two most populated of Norway’s four administrative health regions were included, which are likely to be representative of the whole nation as the study includes both public and private hospitals. We have no reason to believe that systematic errors due to imprecise guidelines would vary between hospitals or regions. Furthermore, the study has a broad approach because it includes data from both primary and revision surgeries, which predominate in the NKLR database. The medical records were thoroughly evaluated by two reviewers, thus ensuring reference data quality. In addition, data were recorded in a predefined form, providing a low risk of information bias.

One weakness of using medical records as a reference standard is that the quality of the records depends on the particular surgeon, and thus information on some variables may be missing or incorrect. If the surgeon does not complete the record directly after surgery, there is also a risk of recall bias for intraoperative details.

Inclusion in the study depended on written consent forms from the patients. Unfortunately, only 89 of the 250 eligible patients (35.6%) returned the consent form. Even though we believe that data from 166 unique surgeries are sufficient to find any systematic errors, other errors might have been discovered if more surgeries had been included.

## Conclusion

In conclusion, our study showed that the NKLR database has high validity, which, when combined with the high registration rate, makes the NKLR a valid resource for future research. However, there is room for improvement, in particular for revision surgery. We wish to emphasize the importance of regular monitoring and validation of the NKLR in the future, to further maintain and improve the quality of the register.

## Supplementary Information

**Additional file 1: Table 1.** Validity of key variables registered in the NKLR for primary reconstructions. Table showing validity of key variables in the NKLR for primary reconstructions.

**Additional file 2: Table 2.** Validity of key variables registered in the NKLR for revision reconstructions. Table showing validity of key variables in the NKLR for revision reconstructions

## Data Availability

The data obtained from the NKLR that support the findings of this study are available from the authors upon reasonable request and with permission of NKLR. The regulations of the Norwegian Data Protection Authority and the Norwegian personal protection laws prohibit the publication of data obtained from the patient journals, which may compromise patients’ privacy/consent.

## Abbreviations

NKLR: Norwegian Knee Ligament Register
ACL: Anterior cruciate ligament
PPV: Positive predictive value
KOOS: The Knee injury and Osteoarthritis Outcome Score
ICRS: International Cartilage Repair Society
PCL: Posterior cruciate ligament
MCL: Medial collateral ligament
LCL: Lateral collateral ligament
PLC: Posterolateral corner
CI: Confidence interval

## References

[1] Kaeding CC, Leger-St-Jean B, Magnussen RA (2017). Epidemiology and diagnosis of anterior cruciate ligament injuries. Clin Sports Med.

[2] Sanders TL, MaraditKremers H, Bryan AJ, Larson DR, Dahm DL, Levy BA (2016). Incidence of anterior cruciate ligament tears and reconstruction: a 21-year population-based study. Am J Sports Med.

[3] Granan L-P, Bahr R, Steindal K, Furnes O, Engebretsen L (2008). Development of a national cruciate ligament surgery registry: the Norwegian National Knee Ligament Registry. Am J Sports Med.

[4] Mohtadi NG, Chan DS (2018). Return to sport-specific performance after primary anterior cruciate ligament reconstruction: a systematic review. Am J Sports Med.

[5] Ardern CL, Webster KE, Taylor NF, Feller JA (2011). Return to sport following anterior cruciate ligament reconstruction surgery: a systematic review and meta-analysis of the state of play. Br J Sports Med.

[6] Engebretsen L, Forssblad M, Lind M (2015). Why registries analysing cruciate ligament surgery are important. Br J Sports Med.

[7] Arts DG, De Keizer NF, Scheffer GJ (2002). Defining and improving data quality in medical registries: a literature review, case study, and generic framework. J Am Med Inform Assoc.

[8] Knatterud GL, Rockhold FW, George SL, Barton FB, Davis CE, Fairweather WR (1998). Guidelines for quality assurance in multicenter trials: a position paper. Control Clin Trials.

[9] Whitney CW, Lind BK, Wahl PW (1998). Quality assurance and quality control in longitudinal studies. Epidemiol Rev.

[10] Ytterstad K, Granan LP, Ytterstad B, Steindal K, Fjeldsgaard KA, Furnes O (2012). Registration rate in the Norwegian Cruciate Ligament Register: large-volume hospitals perform better. Acta Orthop.

[11] Sundbøll J, Adelborg K, Munch T, Frøslev T, Sørensen HT, Bøtker HE (2016). Positive predictive value of cardiovascular diagnoses in the Danish National Patient Registry: a validation study. BMJ Open.

[12] Ytterstad K, Granan LP, Engebretsen L (2011). The Norwegian Cruciate Ligament Registry has a high degree of completeness. Tidsskr Nor Laegeforen.

[13] Visnes H, Kroken G (2021). Annual report NKLR 2020.

[14] Roos EM, Roos HP, Lohmander LS, Ekdahl C, Beynnon BD (1998). Knee Injury and Osteoarthritis Outcome Score (KOOS)–development of a self-administered outcome measure. J Orthop Sports Phys Ther.

[15] Persson A, Kjellsen AB, Fjeldsgaard K, Engebretsen L, Espehaug B, Fevang JM (2015). Registry data highlight increased revision rates for endobutton/biosure HA in ACL reconstruction with hamstring tendon autograft: a nationwide cohort study from the Norwegian Knee Ligament Registry, 2004–2013. Am J Sports Med.

[16] Persson A, Fjeldsgaard K, Gjertsen JE, Kjellsen AB, Engebretsen L, Hole RM (2014). Increased risk of revision with hamstring tendon grafts compared with patellar tendon grafts after anterior cruciate ligament reconstruction: a study of 12,643 patients from the Norwegian Cruciate Ligament Registry, 2004–2012. Am J Sports Med.

[17] Røtterud JH, Sivertsen EA, Forssblad M, Engebretsen L, Årøen A (2016). Effect on patient-reported outcomes of debridement or microfracture of concomitant full-thickness cartilage lesions in anterior cruciate ligament-reconstructed knees: a nationwide cohort study from Norway and Sweden of 357 patients with 2-year follow-up. Am J Sports Med.

[18] Agresti A, Coull BA (1998). Approximate is better than “exact” for interval estimation of binomial proportions. Am Stat.

[19] Gifstad T, Foss OA, Engebretsen L, Lind M, Forssblad M, Albrektsen G (2014). Lower risk of revision with patellar tendon autografts compared with hamstring autografts: a registry study based on 45,998 primary ACL reconstructions in Scandinavia. Am J Sports Med.

[20] Rahr-Wagner L, Thillemann TM, Lind MC, Pedersen AB (2013). Validation of 14,500 operated knees registered in the Danish Knee Ligament Reconstruction Register: registration completeness and validity of key variables. Clin Epidemiol.

[21] Granan LP, Inacio MC, Maletis GB, Funahashi TT, Engebretsen L (2012). Intraoperative findings and procedures in culturally and geographically different patient and surgeon populations: an anterior cruciate ligament reconstruction registry comparison between Norway and the USA. Acta Orthop.

[22] Ulstein S, Bredland K, Aroen A, Engebretsen L, Rotterud JH (2017). No negative effect on patient-reported outcome of concomitant cartilage lesions 5–9 years after ACL reconstruction. Knee Surg Sports Traumatol Arthrosc.

[23] Rotterud JH, Sivertsen EA, Forssblad M, Engebretsen L, Aroen A (2013). Effect of meniscal and focal cartilage lesions on patient-reported outcome after anterior cruciate ligament reconstruction: a nationwide cohort study from Norway and Sweden of 8476 patients with 2-year follow-up. Am J Sports Med.

[24] Granan LP, Bahr R, Lie SA, Engebretsen L (2009). Timing of anterior cruciate ligament reconstructive surgery and risk of cartilage lesions and meniscal tears: a cohort study based on the Norwegian National Knee Ligament Registry. Am J Sports Med.

[25] Salmon LJ, Pinczewski LA, Russell VJ, Refshauge K (2006). Revision anterior cruciate ligament reconstruction with hamstring tendon autograft: 5- to 9-year follow-up. Am J Sports Med.

[26] Wright RW, Huston LJ, Haas AK, Pennings JS, Allen CR, Cooper DE (2021). Association between graft choice and 6-year outcomes of revision anterior cruciate ligament reconstruction in the MARS cohort. Am J Sports Med.

[27] Reverte-Vinaixa MM, Minguell J, Joshi N, Diaz-Ferreiro EW, Duarri G, Carrera L (2014). Revision anterior cruciate ligament reconstruction using tibial or hamstring tendon allografts. J Orthop Surg (Hong Kong).

[28] Di Benedetto P, Di Benedetto E, Fiocchi A, Beltrame A, Causero A (2016). Causes of failure of anterior cruciate ligament reconstruction and revision surgical strategies. Knee Surg Relat Res.

[29] Wilde J, Bedi A, Altchek DW (2014). Revision anterior cruciate ligament reconstruction. Sports Health.

[30] Getelman MH, Friedman MJ (1999). Revision anterior cruciate ligament reconstruction surgery. J Am Acad Orthop Surg.

[31] Redler A, Iorio R, Monaco E, Puglia F, Wolf MR, Mazza D (2018). Revision anterior cruciate ligament reconstruction with hamstrings and extra-articular tenodesis: a mid- to long-term clinical and radiological study. Arthroscopy.

[32] Carson EW, Anisko EM, Restrepo C, Panariello RA, O'Brien SJ, Warren RF (2004). Revision anterior cruciate ligament reconstruction: etiology of failures and clinical results. J Knee Surg.

[33] Brown CH, Carson EW (1999). Revision anterior cruciate ligament surgery. Clin Sports Med.

[34] MARS Group (2020). Predictors of clinical outcome following revision anterior cruciate ligament reconstruction. J Orthop Res.

[35] Kraeutler MJ, Welton KL, McCarty EC, Bravman JT (2017). Revision anterior cruciate ligament reconstruction. J Bone Joint Surg Am.

