# Supplementary material for: Good validity in the Norwegian Knee Ligament Register: assessment of data quality for key variables in primary and revision cruciate ligament reconstructions from 2004 to 2013
Source: BMC Musculoskelet Disord. 2022 Mar 9;23:231. doi: 10.1186/s12891-022-05183-2 (PMC8908681; doi:10.1186/s12891-022-05183-2)
Supplement: Supplementary file 2 — Additional file 2: Table 2. Validity of key variables registered in the NKLR for revision reconstructions. Table showing validity of key variables in the NKLR for revision reconstructions [file 12891_2022_5183_MOESM2_ESM.docx]

**Table 2:** Validity of key variables registered in NKLR for revision reconstructions

|  | **NKLR database** | **Medical record**  **Yes No** | | **Missing in medical record** | **Missing in NKLR** | **PPV% (95% CI)** |
| --- | --- | --- | --- | --- | --- | --- |
| Cartilage lesions | Yes  No | 30  15 | 3  32 | 3 | 0 | 30/33 = 91 (76-98) |
| Meniscal lesions | Yes  No | 27  0 | 1  54 | 1 | 0 | 27/28 = 96 (80-100) |
| Diagnosis of ACL lesion | Yes  No | 66  16 | 0  0 | 0 | 0 | 66/66 = 100 |
| Other ligament lesions^†^ | Yes  No | 5  0 | 0  78 | 0 | 0 | 5/5 = 100 |

|  | **Result of validation** | **Number of cases** | **Missing in medical record** | **Missing in NKLR** | **PPV% (95% CI)** |
| --- | --- | --- | --- | --- | --- |
| OP date^‡^ | Identical  Not identical | 81  2 | 0 | 0 | 81/83 = 98 (91-100) |
| Graft choice^§^ | Identical  Not Identical | 83  0 | 0 | 0 | 83/83 = 100 |
| Method of fixation in femur | Identical  Not identical  Partly identical^¶^ | 56  1  0 | 0 | 2 | 56/57 = 98 (90-100) |
| Method of fixation in tibia | Identical  Not Identical  Partly identical^¶^ | 54  1  0 | 0 | 4 | 54/55 = 98 (89-100) |
| Cause of revision | Identical  Not identical  Partly identical^¶^ | 63  4  3 | 0 | 13 | 63/67 = 94 (85-98) |
| Activity at the time of injury | Identical  Not identical | 43  39 | 1 | 0 | 43/82 = 52 (42-63) |
| Date of injury | Identical  Not identical | 30  46 | 5 | 2 | 30/76 = 39 (29-51) |

**Footnotes:** ^†^ Includes PCL, MCL, LCL and PLC. ^‡^ Date of operation has to be identical (exact date) to be regarded as correct. ^§^ Includes BPTB, hamstring, other types of grafts and no graft chosen. ^¶^ Patients with both ACL and PCL lesions, but only one of the lesions registered in the NKLR, are labelled “partly identical” and are not taken into account when the PPVs are computed.

**Abbreviations:** NKLR, Norwegian Knee Ligament Register; PPV, positive predictive value; CI, confidence interval; ACL, anterior cruciate ligament; OP, operation; PCL, posterior cruciate ligament; MCL, medial collateral ligament; LCL, lateral collateral ligament; PLC, posterolateral corner; BPTB, bone-patellar tendon-bone graft.
